# Improved Sensitivity of a Taste Sensor Composed of Trimellitic Acids for Sweetness

**DOI:** 10.3390/molecules29235573

**Published:** 2024-11-25

**Authors:** Tatsukichi Watanabe, Sojiro Kumura, Shunsuke Kimura, Kiyoshi Toko

**Affiliations:** 1 Graduate School and Faculty of Information Science and Electrical Engineering, Kyushu University, 744 Motooka, Nishi-ku, Fukuoka 819-0395, Japan; kumura.sojiro.145@s.kyushu-u.ac.jp; 2 Research and Development Center for Five-Sense Devices, Kyushu University, 744 Motooka, Nishi-ku, Fukuoka 819-0395, Japan; s.kimura@nakamura-u.ac.jp; 3 Faculty of Nutritional Sciences, Nakamura Gakuen University, 5-7-1 Befu, Jonan-ku, Fukuoka 814-0198, Japan; 4 Food and Health Innovation Center, Nakamura Gakuen University, 5-7-1 Befu, Jonan-ku, Fukuoka 814-0198, Japan; 5 Institute for Advanced Study, Kyushu University, 744 Motooka, Nishi-ku, Fukuoka 819-0395, Japan; 6 Graduate School of Nutritional Sciences, Nakamura Gakuen University, 5-7-1 Befu, Jonan-ku, Fukuoka 814-0198, Japan

**Keywords:** taste sensor, lipid/polymer membrane, sweetness, membrane potential

## Abstract

Currently, lipid/polymer membranes are used in taste sensors to quantify food taste. This research aims to improve sweetness sensors by more selectively detecting uncharged sweetening substances, which have difficulty obtaining a potentiometric response. Lipid/polymer membranes with varying amounts of tetradodecylammonium bromide (TDAB) and 1,2,4-benzene tricarboxylic acid (trimellitic acid) were prepared. The carboxyl groups of trimellitic acid bind metal cations, and the sweetness intensity is estimated by measuring the potential change, as a sensor response, when these cations are complexed with sugars. This research showed that the potential of a sensor using the membrane with enough trimellitic acid in a sucrose solution remained constant, regardless of TDAB amounts, but the potential in the tasteless, so-called reference solution, depended on TDAB. By optimizing the content of TDAB and trimellitic acid, a sensor response of −100 mV was achieved, which is over 20% more sensitive than a previous sensor. This sensor also demonstrated increased selectivity to sweetness, with similar interference from other tastes (saltiness, sourness, umami, and bitterness) compared to previous sensors. As a result, the sensitivity to sweetness was successfully improved. This result contributes to the development of novel sensors, further reducing the burden on humans in quality control and product development.

## 1. Introduction

Sweetness, one of the five basic tastes, is mainly presented by sugars and other substances, and is used by humans and many other vertebrates as a signal of energy source [1]. In addition to sugars (e.g., sucrose), compounds with diverse chemical structures and sizes, such as sugar alcohols (e.g., mannitol), sulfonyl amides (e.g., sodium saccharin), d-amino acids (e.g., d-tryptophan), peptides (e.g., aspartame), and proteins (e.g., thaumatin) are known to have sweet taste. Only heterodimeric receptors (T1R2 + T1R3) respond to sweetening substances, but there is no chemical structure or physicochemical property common only to sweetening substances [2,3]. The AH-B theory, in which the hydrogen donor is represented as AH and the hydrogen acceptor as B, is one of the most widely accepted models, but models that can account for structural features common only to sweetening substances without exception do not exist [3,4,5,6]. It is now supplemented that the hydrophobic site X must be properly aligned with AH and B [7]. The sweetening substances are accepted by electrostatic and hydrophobic interactions between the Venus flytrap domains of T1R2 and T1R3, which are sweetness receptors in humans, and various glucophores (hydroxy groups, oxygen atoms, hydrocarbon groups, nonpolar residues, etc.) of various sweetening substances [8,9,10].

Brix meter (refractometer) and NIR or FT-IR techniques have been developed and commercialized as methods to evaluate the sweetness of foods [11,12,13,14]. However, these methods mainly estimate the amount of sugar, an uncharged sweetening substance, and cannot measure all types of sweetening substances.

Electronic tongue and taste sensors, a type of chemical sensor, have been developed and are used for the development of new products in the food and pharmaceutical industries and for quality control of existing products. The taste sensor developed in Japan has a lipid/polymer membrane on the sensor electrode and can quantify the quality and intensity of taste by measuring the potential of the sensor in a taste sample. Taste sensors mimic human taste perception, with several different membranes designed to respond selectively to different tastes, and then can discriminate and quantify the five basic tastes of sweetness, saltiness, sourness, umami, and bitterness; this property is called global selectivity [15,16,17,18,19,20,21,22,23,24,25,26,27,28]. It is difficult to measure sweetness using only one sensor membrane because sweetening substances are broadly classified into three groups according to the charges they carry (no charge, positive charge, and negative charge). Therefore, three types of sweetness sensors have been developed for each type of charge [29,30,31]. A sweetness sensor for uncharged sweetening substances (mainly sugars) has been commercialized, but this sensor responds not only to sweetness, but also to saltiness [29], and its selectivity for sweetness is not satisfactory.

In previous research, we reported that the sensitivity of previous sweetness sensors can be improved by changing the potassium ions in the conditioning solution, used to clean the sensor membrane during measurement, to sodium ions [32]. This is because the optimum ionic radius of metal ions that form complexes in the ax-eq-ax arrangement, which consists of three hydroxy groups in the six-membered ring structure commonly possessed by sugars and is suitable for complex formation with metal ions, is 0.1 nm, and among monovalent metal ions, the ionic radius of the sodium ion is the most suitable [33].

However, the sensitivity to sweetness obtained in the research [32] was still not sufficient, and hence the improvement of sensitivity was eagerly desired for practical application. Given that the composition of the previous sweetness sensor membranes had not been investigated in detail, this research aimed to further develop the sweetness sensor for practical use by investigating the detailed composition of the membrane. In this research, we investigated the effects of lipid tetradodecylammonium bromide (TDAB) and modifier 1,2,4-benzene tricarboxylic acid (trimellitic acid) content in the lipid/polymer membrane on the sensor sensitivity by taking account of the binding of metal cations with the carboxy groups of trimellitic acid followed by binding with sugars. As a result, a sweetness sensor with a response value exceeding that of previous sweetness sensors was realized, whereas it exhibited the same level of response to taste substances other than sweetness as the previous sensors, signifying the success of improvement of the selectivity for sweetness. Given this, the sensor developed in this research will contribute to product development and quality control of sweet-tasting products.

## 2. Results and Discussion

### 2.1. Effect of Lipid and Modifier Content in Sweetness Sensor Membrane on Reference Potential and Relative Value

**Figure 1:**
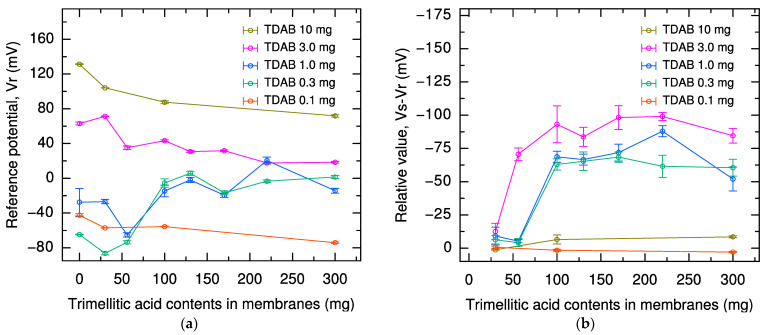
(**a**) Reference potential for sensors with 0.1, 0.3, 1.0, 3.0, and 10 mg TDAB and 0, 30, 56, 100, 130, 170, 220, and 300 mg trimellitic acid, respectively. (**b**) Relative value in 1000 mM sucrose solution for sensors with 0.1, 0.3, 1.0, 3.0, and 10 mg TDAB and 30, 56, 100, 130, 170, 220, and 300 mg trimellitic acid, respectively. The error bar indicates the standard deviations (SD) of the data, *n* = 4 (electrode) × 3 (rotation) = 12 values.

First, the effects of lipid and modifier content in the lipid/polymer membrane on the reference potential in the sweetness sensor were investigated. The reference potentials of sensors with various lipid and modifier contents are shown in Figure 1a. The potential measured in the reference solution (0.3 mM tartaric acid and 30 mM KCl) was named the reference potential (*V*r).

As the amount of lipid TDAB in the membrane increased, the reference potential of the sensor tended to become more positive, with a difference of about 100 mV between sensors containing 0.1 mg and 10 mg of TDAB, regardless of the amount of modifier trimellitic acid. This is attributed to the fact that quaternary ammonium ions derived from TDAB positively charge the membrane [34,35,36,37,38].

Focusing on the relationship between trimellitic acid content and reference potential, the reference potential shifted positively by 63 mV and 19 mV for TDAB 0.3 and 1.0 mg membranes, respectively, with an increase in the trimellitic acid content from 0 to 100 mg. This is due to the binding of sodium ions to the carboxy groups of trimellitic acid and the phosphate groups in the impurities of the plasticizer DOPP, as discussed [39] and demonstrated [40], during the cleaning process of immersion in the conditioning solution.

On the other hand, for membranes with TDAB of 3.0 and 10 mg, the reference potentials decreased by 20 mV and 42 mV, respectively, as the trimellitic acid content increased in the range of 0 mg to 100 mg. These membranes have a positive reference potential in the absence of trimellitic acid, i.e., the membrane surface is positively charged, so that sodium ions cannot approach the membrane when immersed in the conditioning solution. Therefore, the carboxy groups of trimellitic acid could not bind enough sodium ions, and the carboxy groups that did not bind sodium ions existed as charged substances on the membrane surface, so the sensor potential in these membranes shifted negatively as the trimellitic acid content increased.

Next, we investigated the effect of lipid and modifier content in the lipid/polymer membrane on the relative value (*V*s – *V*r). This relative value was defined by the difference between *V*s and *V*r when the potential in the sample solution (*V*s) was measured, and this value represents the sensor sensitivity. The relative values of 1000 mM sucrose solution measured using the sensors with various lipid and modifier contents are shown in Figure 1b. 

Measurements using the sensor membranes with 0.1 and 10 mg of TDAB did not function as a sucrose-responsive sensor, as found in Figure 1b. This is due to the excess of positive and negative loading on the surface of the respective membranes, which results in small changes in membrane potential due to changes in the charge state of the membrane surface [38]. Another reason may be that in the sensor containing 10 mg of TDAB, sodium ions, which are important in the sucrose detection mechanism, did not bind to the membrane during immersion in the conditioning solution, as mentioned above.

On the other hand, in the sensor membranes containing the intermediate concentrations, i.e., 0.3, 1.0, and 3.0 mg of TDAB, the responses to sucrose appeared clearly. Furthermore, the effect of trimellitic acid content on sensor sensitivity, i.e., the relative value, is important; for example, in the case of the 3.0 mg TDAB sensor membrane, the relative value increased significantly from −13 mV to −93 mV with an increase in the trimellitic acid from 30 mg to 100 mg. However, when the trimellitic acid content was increased above 100 mg, this increase in sensitivity plateaued for all TDAB contents.

Focusing on the relationship between the TDAB content and the relative value in the range of sufficient sensor sensitivity, where the trimellitic acid content is 100 mg or more, the sensitivity was improved by increasing the TDAB content from 0.1 mg to 3.0 mg. This can be attributed to the positive shift of *V*r with increasing TDAB content in the sensor (Figure 1a). Since the relative value is given by (*V*s – *V*r), it is essential to increase the difference between *V*s and *V*r to obtain a sensor with high sensitivity.

**Figure 2:**
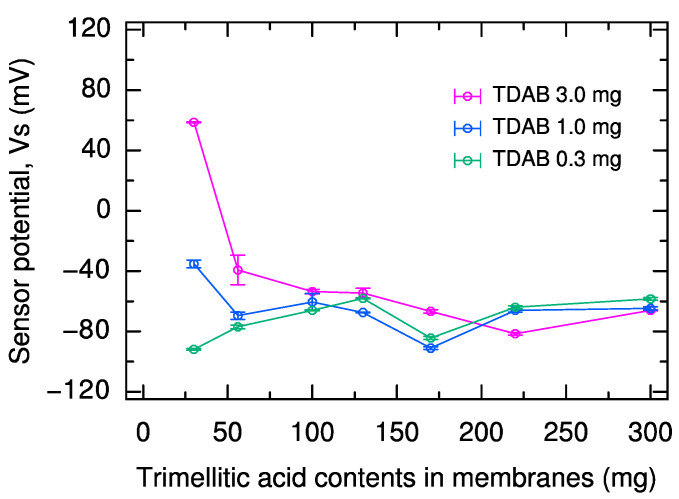
Sensor potential in 1000 mM sucrose solution for sensors with 0.3, 1.0, and 3.0 mg TDAB and 30, 56, 100, 130, 170, 220, and 300 mg trimellitic acid, respectively. The error bar indicates the SD of the data, *n* = 4 (electrode) × 3 (rotation) = 12 values.

In the *V*s of these sensors (Figure 2), *V*s was in the range of −70 ± 20 mV with only minor fluctuations, regardless of TDAB or trimellitic acid content, in the range of 100 mg or more trimellitic acid. Thus, the positive shift in *V*r increased the sensor sensitivity, i.e., (*V*s – *V*r). The reason why *V*s is constant with sufficiently high trimellitic acid content is that the loading charge derived from the carboxy group of trimellitic acid is excessive in the sucrose solution, and the effect of the trimellitic acid and TDAB content on *V*s is eliminated. Furthermore, under such a high concentration condition as 1000 mM sucrose solution, the sodium ions bound on the sensor membrane in the conditioning solution are considered to be completely removed by the sucrose. Therefore, there is no need to consider the effect of the remaining sodium ions on the membrane, which function as a positive charge and shift *V*s positively.

These results indicate that increasing the trimellitic acid content in the membrane up to 100 mg significantly improves the sensor sensitivity, i.e., (*V*s – *V*r), whereas increasing the content beyond 100 mg does not contribute to the sensitivity improvement because *V*s has already reached its lower limit. Furthermore, increasing the TDAB content shifts *V*r positively and thus contributes to a large relative value. It is also clear that under conditions of excess TDAB content, such as 10 mg, the adsorption of sodium ions is inhibited by the strong positive charge on the membrane surface, so that no change in potential occurs due to desorption of sodium ions in the sucrose solution, and the sensitivity is significantly reduced. Summary of shifts in reference potential, relative values, and *V*s due to TDAB and trimellitic acid content are shown in Table 1.

**Figure 3:**
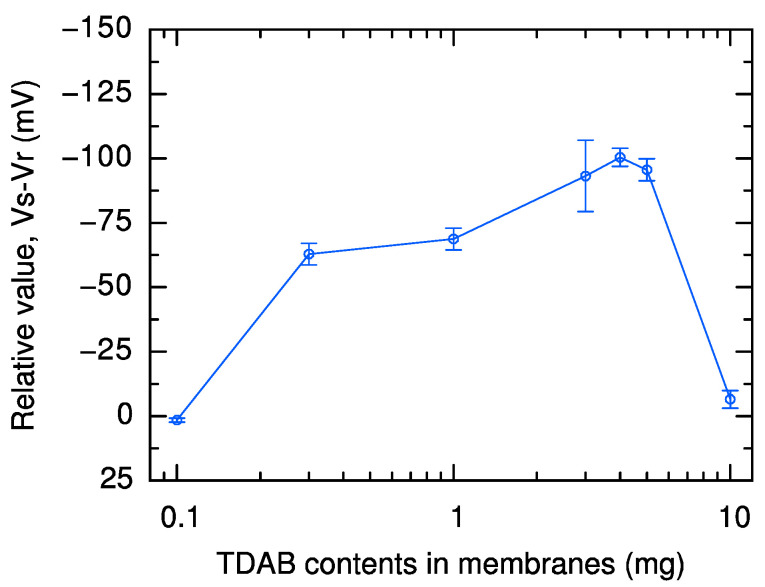
Relative value in 1000 mM sucrose solution for sensors with 100 mg trimellitic acid and 0.1, 0.3, 1.0, 3.0, 4.0, 5.0, and 10 mg TDAB. The error bar indicates the SD of the data, *n* = 4 (electrode) × 3 (rotation) = 12 values.

Figure 3 shows the relative values for the TDAB content from 0.1 mg to 10 mg with 100 mg trimellitic acid. In addition to the sensors shown so far, two sensors with TDAB content of 4.0 mg or 5.0 mg were tested to search for conditions with even higher sensor output. The sensor with 4.0 mg TDAB and 100 mg trimellitic acid is over 20% more sensitive than the −80 mV relative value of the sensor in the previous research (1.0 mg TDAB and 100 mg trimellitic acid) [32] that used a similar mechanism. The sensor is also over 50% more sensitive than the −65 mV relative value of a commercial sweetness sensor, GL1 [29]. The most significant factor in the sensor response we developed in this research, which was greater than the sensor response obtained in previous research, was the positive shift in the reference potential due to the increased TDAB content.

### 2.2. Response to Five Basic Taste Samples

**Figure 4:**
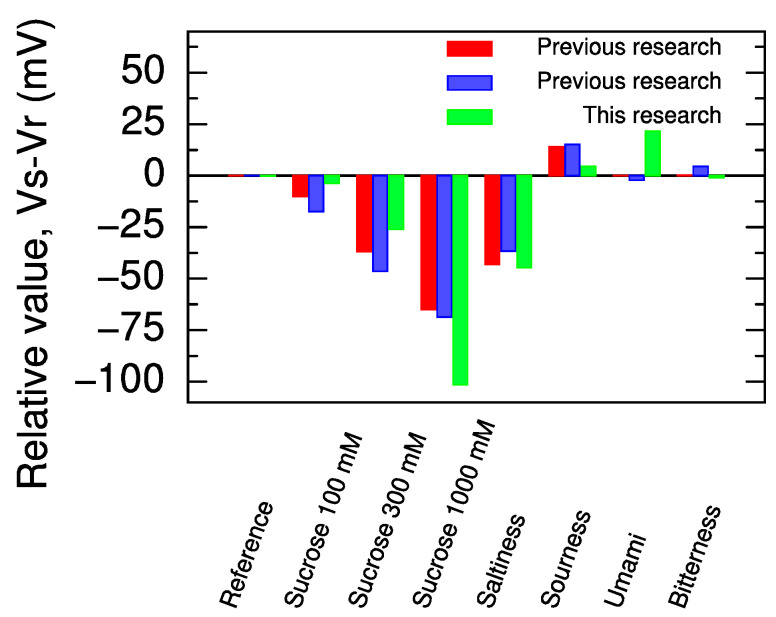
Comparison of responses in five basic taste samples for the sensor developed in this research (4 mg TDAB and 100 mg trimellitic acid) and the sensor (1 mg TDAB and 100 mg trimellitic acid) presented in previous research [29,32]. In previous research (red) [29], the conditioning solution during the measurement contained potassium ions, and in previous research (blue) [32], the conditioning solution contained sodium ions.

Finally, we examined the response to the five basic taste samples (sweetness: 100, 300, and 1000 mM sucrose solutions; saltiness: 0.3 mM tartaric acid and 300 mM KCl solution; sourness: 3.0 mM tartaric acid and 30 mM KCl solution; umami: 10 mM MSG solution; and bitterness: 0.1 mM quinine hydrochloride dihydrate solution) by the sweetness sensor with 4.0 mg TDAB and 100 mg trimellitic acid. The compositions of these samples are summarized in Table 2. The results are shown in Figure 4 together with the results of the basic five tastes measured by the sensor membranes (1.0 mg TDAB and 100 mg trimellitic acid) in previous research [29,32].

The response of the sensor membrane in this research to 1000 mM sucrose solution was more than 30 mV greater than the results of the previous research. Furthermore, the response to the other samples than sweetness, which interfere with the sweetness measurements, was comparable to the response values in the previous research. In other words, the sensor developed in this research (4 mg TDAB and 100 mg trimellitic acid) was able to achieve greater sensitivity to sweetness than previous sensors while keeping sensitivity to other tastes at about the same level as previous sensors. These results indicate that the sweetness sensor optimized in this research has a wide dynamic range for sweetness, while the undesirable response to non-sweet samples is suppressed to the same level as that of the previous sweetness sensor.

## 3. Materials and Methods

### 3.1. Reagent

**Figure 5:**
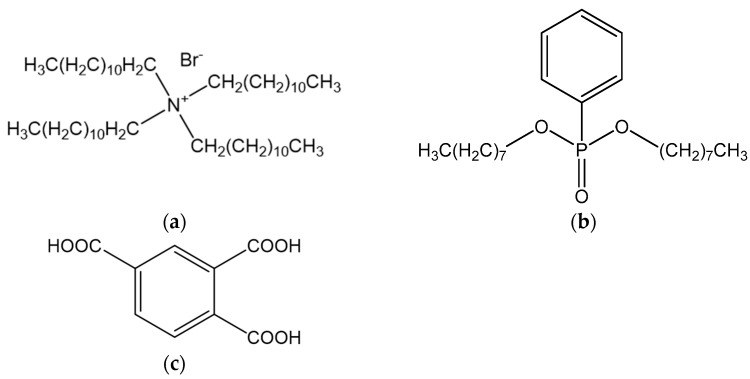
Structure of membrane components (**a**) TDAB, (**b**) DOPP, and (**c**) trimellitic acid.

Tetradodecylammonium bromide (TDAB, Sigma-Aldrich Japan G.K., Tokyo, Japan) was used as a positively charged lipid of the membrane. Polyvinyl chloride (PVC, Fujifilm Wako Pure Chemicals Corporation, Osaka, Japan) was used as a polymer support. The compound 1,2,4-Benzene tricarboxylic acid (trimellitic acid, Tokyo Chemical Industry Co., Tokyo, Japan) was used as a modifier. Di-n-octyl phenylphosphonate (DOPP, Fujifilm Wako Pure Chemicals Corporation, Osaka, Japan) was used as a plasticizer for the membrane. Tetrahydrofuran (THF, Sigma-Aldrich Japan G.K., Tokyo, Japan) was used as the organic solvent to prepare the solution for membrane formation. Figure 5 shows the structure of the three materials comprising the membrane. Sodium hydroxide (NaOH) was purchased from Fujifilm Wako Pure Chemicals Corporation, Osaka, Japan. Sodium chloride (NaCl), potassium chloride (KCl), sucrose, monosodium L-glutamate monohydrate (MSG), and quinine hydrochloride dihydrate were purchased from Kanto Chemical Company, Tokyo, Japan. All reagents were used without purification.

### 3.2. Solution Preparation

Aqueous solutions containing tartaric acid and KCl at concentrations of 0.3 mM and 30 mM, respectively, were prepared as reference solutions for the measurement of reference potentials. For the sample solutions, sucrose was dissolved in the reference solution at concentrations of 100, 300, and 1000 mM as the sweetness sample. Aqueous solutions containing tartaric acid and KCl at concentrations of 0.3 mM and 300 mM, respectively, were prepared as the saltiness sample, and tartaric acid and KCl at concentrations of 3.0 mM and 30 mM, respectively, as the sourness sample. MSG and quinine hydrochloride dihydrate were dissolved in the reference solution to concentrations of 10 mM and 0.1 mM, respectively, as the umami and bitterness samples. The conditioning solution used to clean the sensor membranes in the measurement was prepared at a concentration of 10 mM NaOH and 100 mM NaCl (solvent/water:EtOH = 7:3).

### 3.3. Formation of Lipid/Polymer Membrane and Fabrication of Sensor Electrodes

**Figure 6:**
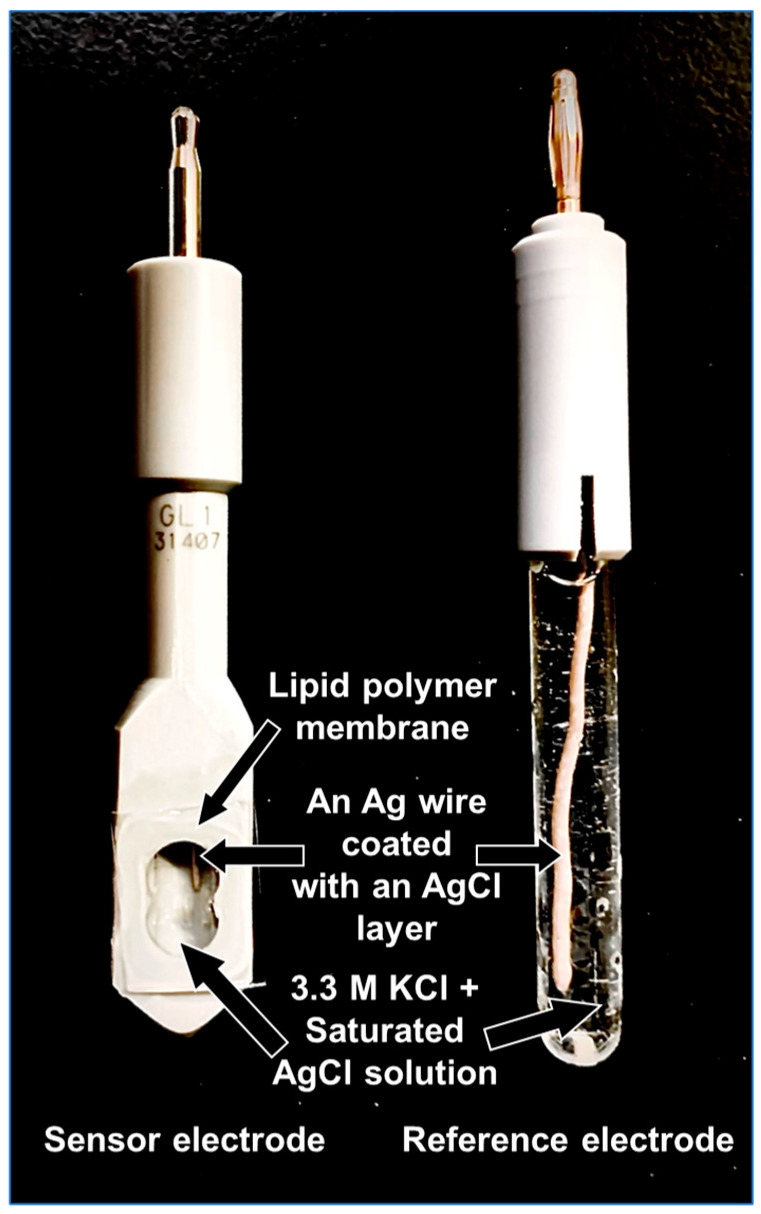
Structure of taste sensor electrode and reference electrode.

A content range of more than two orders of magnitude was selected based on 1 mg of TDAB and 100 mg of trimellitic acid used in the sweetness sensor in previous research [29,32], to investigate the effect of TDAB and trimellitic acid content on the reference potential and relative values. The contents of TDAB and trimellitic acid ranged from 0.1 mg to 10 mg and 0 mg to 300 mg, respectively, and these, plus 1.5 mL of DOPP and 800 mg of PVC, were dissolved in 10 mL of THF. The prepared gel solution was then poured into a 90 mm petri dish to volatilize the THF. The thickness of the finished membrane was 0.35 mm, and the volume of the membrane was 45 mm × 45 mm × π × 0.35 mm. The membranes were cut into appropriate sizes and attached to hollow sensor probes with an adhesive consisting of PVC and THF. Finally, an internal solution (3.33 M KCl, saturated AgCl solution) was injected, and a Ag wire coated with a AgCl layer was inserted. Figure 6 (left) shows the structure of the sensor electrode; four sensors of equal quality were fabricated and measured from a single petri dish.

### 3.4. Measurement Procedure and Response Mechanism of Taste Sensors

A taste recognition device (TS-5000Z, Intelligent Sensor Technology, Inc., Kanagawa, Japan) was used to measure the sensor potential. The measurement procedure in this research followed that of a previous report [32]. The reference electrode used a Ag wire electrode coated with a AgCl layer and 3.33 M KCl, saturated AgCl solution as the internal solution (Figure 6 (right)). First, as a cleaning process, the sensor was immersed in the conditioning solution, and then the potential in the reference solution was measured to determine the reference potential (*V*r). The potential in the sample solution (*V*s) was then measured, and the difference between *V*s and *V*r (*V*s − *V*r) was taken as the relative value, i.e., the sensor’s response value in the various solutions. The above procedure was repeated for each of the four sensors in five cycles. Since the reference potential showed a stable value from the third cycle in the cycle of measurements, the data from the third to the fifth cycle of each of the four sensors (12 data in total) were used to calculate the relative values and standard deviations.

**Figure 7:**
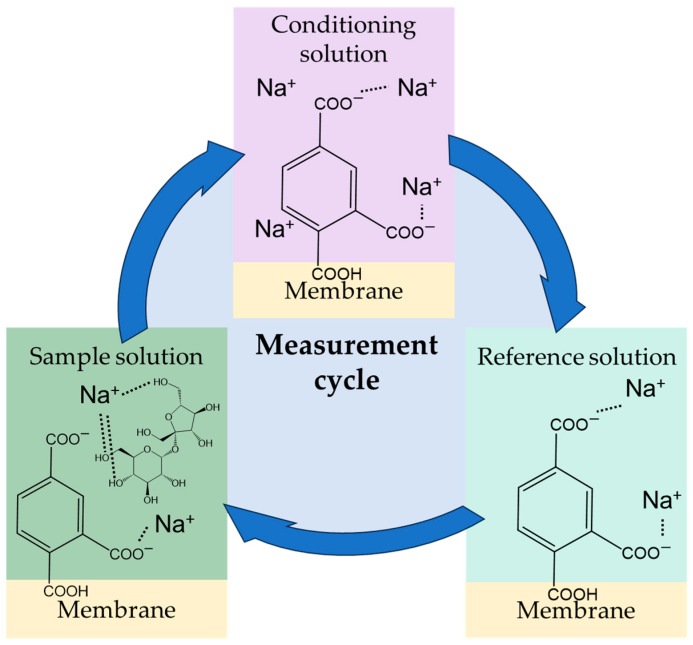
Measurement cycle and state of binding of sodium ions to trimellitic acid, a modifier, on the surface of the sensor membrane in the conditioning solution, reference solution, and sample solution.

It has been reported that the reference potential (*V*r) shifts positively with repeated cycles of cleaning process [32]. This is because each time the sensor membrane is cleaned with the conditioning solution during the measurement cycle, the sodium ions in the solution bind to the carboxy groups of the trimellitic acid in the membrane, and this state is retained to measure *V*r in the reference solution. Some of the sodium ions bind to the carboxy groups in the conditioning solution and form metal complexes with sucrose in the sample solution (Figure 7). The sensor potential (*V*s) is the potential that is negatively shifted from *V*r due to the desorption of sodium ions adsorbed the membrane in this process. Thus, the relative value can be used as an indicator of sweetness intensity.

## 4. Conclusions

The sensitivity to sweetness obtained previously using taste sensors was not satisfactory, and hence this research aimed at improving sensitivity. We investigated the effects of the amount of lipid in the membrane and the amount of trimellitic acid as modifier on the reference potential and relative value of a sweetness sensor that responds to sugar, a representative sweetness substance. The results showed that increasing the trimellitic acid content in the membrane up to 100 mg significantly improved the relative value, while increasing the content beyond that content did not contribute to an increase in the relative value because *V*s reached a lower limit. Furthermore, increasing the content of TDAB resulted in a positive shift in *V*r, which in turn contributed to a large relative value. Based on this research, we have successfully developed a taste sensor that exhibits a sweetness response above −100 mV to 1000 mM sucrose, while keeping the undesirable response to non-sweet samples at the same level as previous sweetness sensors. These results will contribute to product development and quality control of sweet-tasting products, as well as to the development of sensor membranes with a wider concentration range that can be measured.

## Figures and Tables

**Table 1:** Summary of shifts in reference potential, relative value, and *V*s due to TDAB and trimellitic acid content.

Reference Potential	Relative Value	*V*s
Positive shift with increasing TDAB content	Increasing up to 3 mg TDAB, or up to 100 mg trimellitic acid	Only minor fluctuations above 100 mg trimellitic acid

**Table 2:** Composition of five basic tastes sample based on reference solution (30 mM KCl and 0.3 mM tartaric acid).

Sample	Composition	Concentration
Reference	KCl and Tartaric acid	30 mM and 0.3 mM, respectively
Sweetness	Sucrose	100, 300, 1000 mM
Saltiness	KCl	300 mM
Sourness	Tartaric acid	3 mM
Umami	Monosodium L-glutamate (MSG)	10 mM
Bitterness	Quinine hydrochloride	0.1 mM

## Data Availability

The data presented in this research are available on request.
